# Controlling Topology within Halogen-Bonded Networks by Varying the Regiochemistry of the Cyclobutane-Based Nodes

**DOI:** 10.3390/molecules26113152

**Published:** 2021-05-25

**Authors:** Taylor J. Dunning, Daniel K. Unruh, Eric Bosch, Ryan H. Groeneman

**Affiliations:** 1 Department of Biological Sciences, Webster University, St. Louis, MO 63119, USA; taylordunning65@webster.edu; 2 Department of Chemistry and Biochemistry, Texas Tech University, Lubbock, TX 79409, USA; daniel.unruh@ttu.edu; 3 Department of Chemistry, Missouri State University, Springfield, MO 65897, USA; EricBosch@MissouriState.edu

**Keywords:** halogen bonding, crystal engineering, organic solid state, topology

## Abstract

The formation of a pair of extended networks sustained by halogen bonds based upon two regioisomers of a photoproduct, namely *rctt*-1,3-bis(4-pyridyl)-2,4-bis(phenyl)cyclobutane (*ht*-PP) and *rctt*-1,2-bis(4-pyridyl)-3,4-bis(phenyl)cyclobutane (*hh*-PP), that have varied topology is reported. These networks are held together via I⋯N halogen bonds between the photoproduct and the halogen-bond donor 1,4-diiodoperchlorobenzene (C_6_I_2_Cl_4_). The observed topology in each solid is controlled by the regiochemical position of the halogen-bond accepting 4-pyridyl group. This paper demonstrates the ability to vary the topology of molecular networks by altering the position of the halogen bond acceptor within the cyclobutane-based node.

## 1. Introduction

Halogen bonding, the interaction between an electrophilic region on a halogen atom within a molecule and a nucleophilic region on a different atom, is a well-established and recognized non-covalent interaction [1]. Halogen bonding interactions have been one of the main driving forces in the formation of numerous organic solids [2,3]. In particular, the formation of extended molecular networks held together by these interactions continues to be an active area of research for crystal engineers. As in all networks, the careful selection of both the node and the linker is critical in the overall design of the framework along with the desired topology [4]. The incorporation of nodes based upon cyclobutane photoproducts, generated in the solid state via the [2 + 2] cycloaddition reaction, continues to yield diverse molecular solids. The photoproduct *rctt*-tetrakis(4-pyridyl)cyclobutane (TPCB) has been shown to act as a four-connecting node in the formation of different multi-dimensional extended molecular solids based upon halogen bonding interactions [5,6].

**Scheme 1:**
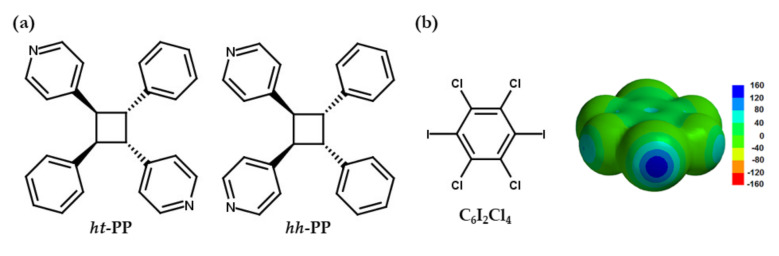
Components of the halogen-bonded networks: (**a**) halogen-bond acceptors *ht*-PP and *hh*-PP with indicated regiochemistry around the cyclobutane-based nodes and (**b**) the halogen-bond donor C_6_I_2_Cl_4_ with molecular electrostatic potential plot.

The ability to modify the pendant groups off of the central cyclobutane ring is achieved by simply changing the olefin-containing molecule that undergoes the photodimerization reaction [7]. This synthetic control over these pendant groups allows chemists to either incorporate or remove organic functionality within the targeted photoproduct. In particular, the position and number of halogen-bond donor and acceptor sites can be programmed into these photoproducts. As a result, these intentional designed photoproducts allow chemists to ultimately control the overall structure and topology of the network. To this end, the ability to replace some of the 4-pyridyl groups on TPCB with phenyl groups, which are unable to accept halogen bonds, will alter the structure and resulting topology of the solid. This substitution of aromatic rings off of the cyclobutane is accomplished by simply photoreacting 4-stilbazole (4-SB) with an appropriate template to yield either regioisomer of the photoproduct, namely *rctt*-1,3-bis(4-pyridyl)-2,4-bis(phenyl)cyclobutane (*ht*-PP) [8] and *rctt*-1,2-bis(4-pyridyl)-3,4-bis(phenyl)cyclobutane (*hh*-PP) (Scheme 1a) [9].

Herein, we report a pair of halogen-bonded networks based upon the regioisomers *ht*-PP and *hh*-PP along with 1,4-diiodoperchlorobenzene (C_6_I_2_Cl_4_) (Scheme 1b) resulting in a linear and zigzag topology, respectively. We have recently demonstrated that C_6_I_2_Cl_4_ is a reliable I⋯N halogen-bond donor that also participates in face-to-face π⋯π stacking, thereby aligning alkene-based reactant molecules to undergo a [2 + 2] photoreaction in the organic solid state [8,10]. The σ-hole within C_6_I_2_Cl_4_ was determined to be 145.7 kJ/mol based upon a molecular electrostatic potential calculation (Scheme 1b) [10]. With this value, C_6_I_2_Cl_4_ is well within the range of a reliable halogen-bond donor [11]. The different positions of the 4-pyridyl groups on these photoproducts directly influenced the resulting topology. This work presented here demonstrates the ability to control the overall crystal structure of these networks by simply changing the location of the halogen-bond acceptor sites on the cyclobutane-based node.

## 2. Results and Discussion

### 2.1. X-ray Crytstal Strucutre of the Linear Topology within (C_6_I_2_Cl_4_)·(ht-PP)

**Figure 1:**
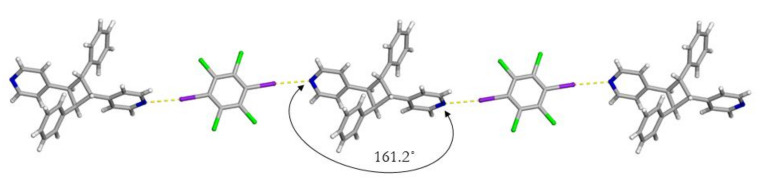
X-ray structure of (C_6_I_2_Cl_4_)·(*ht*-PP) illustrating the I*⋯*N halogen bonds that generate the observed linear topology due to the 1,3-position of the 4-pyridyl groups around the cyclobutane ring. Grey ball-C atom, white ball-H atom; green ball-Cl atom; purple ball-I atom; blue ball-N atom.

The components of (C_6_I_2_Cl_4_)·(*ht*-PP) crystallize in the triclinic space group Pī (Table 1), where a single molecule of each are found in the asymmetric unit. Akin to TPCB, the photoproduct *ht*-PP has four radially splayed aromatic rings coming off the central cyclobutane ring. The observed *rctt*-stereochemistry in *ht*-PP is realized due to the position of the reactant molecules before photoreaction. As a consequence of this stereochemistry, these pendant aromatic rings generate two acute (71.1 and 74.2°) and two obtuse (99.0 and 121.4°) bond angles measured from the centroids of both the aromatic and cyclobutane rings (Figure S1a). As expected, C_6_I_2_Cl_4_ forms I⋯N halogen bonds (I⋯N 2.838(3) and 2.871(3) Å; C-I⋯N 170.5(1) and 175.0(1)°) with the 4-pyridyl rings on *ht*-PP, which generates a one-dimensional linear chain (Figure 1). Since, the 4-pyridyl groups within *ht*-PP are found in the 1,3-position on the cyclobutane, the photoproduct acts as a linear node due to the divergent positions of the pyridine rings with an observed bond angle of 161.2°.

**Figure 2:**
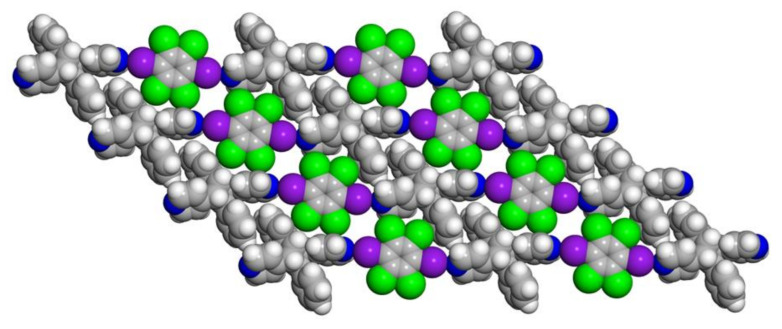
X-ray structure of (C_6_I_2_Cl_4_)·(*ht*-PP) illustrating the packing of neighboring linear chains sustained by Cl⋯π and offset π⋯π stacking interactions. Grey ball-C atom, white ball-H atom; green ball-Cl atom; purple ball-I atom; blue ball-N atom.

These linear chains interact with the nearest neighboring chains by both Cl*⋯*π and offset π⋯π stacking interactions (Figure 2). In particular, all of the chlorine atoms on C_6_I_2_Cl_4_ are found to interact with two pyridyl rings (Cl⋯π 3.31 and 3.70 Å) and two phenyl rings (Cl⋯π 3.53 and 3.75 Å) measured from the chlorine atom to the centroid of the aromatic ring [12]. These Cl⋯π interactions are found to occur between C_6_I_2_Cl_4_ and *ht*-PP within the obtuse angles of the aromatic rings. In contrast, the offset π⋯π stacking interactions (π⋯π 4.51 Å) occur between neighboring phenyl groups and are located within the acute bond angles around the cyclobutane rings.

### 2.2. X-ray Crytstal Strucutre of the Zigzag Topology within 3(C_6_I_2_Cl_4_)·2(hh-PP)·4(toluene)

**Figure 3:**
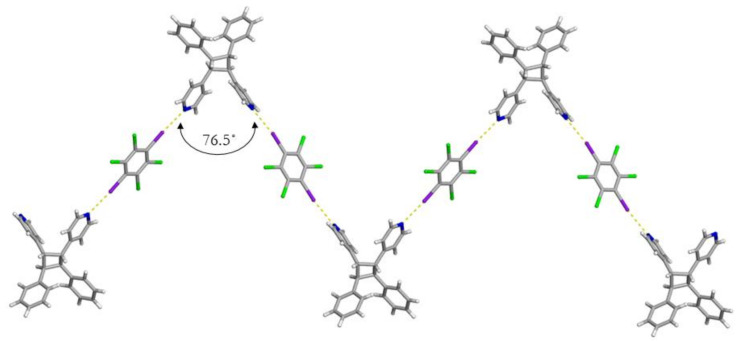
X-ray structure of 3(C_6_I_2_Cl_4_)·2(*hh*-PP)·4(toluene) illustrating the I*⋯*N halogen bonds that result in the observed zigzag topology due to the 1,2-position of the 4-pyridyl groups around the cyclobutane ring. Grey ball-C atom, white ball-H atom; green ball-Cl atom; purple ball-I atom; blue ball-N atom.

The components of 3(C_6_I_2_Cl_4_)·2(*hh*-PP)·4(toluene) also crystallize in the triclinic space group Pī (Table 1). Unlike (C_6_I_2_Cl_4_)·(*ht*-PP), the asymmetric unit of this solid contains a single molecule of *hh*-PP along with three half occupied C_6_I_2_Cl_4_ and two toluene molecules. After refinement of the diffraction data, both a molecule of C_6_I_2_Cl_4_ and toluene were found to be disordered over two positions. Similar to other photoproducts, *hh*-PP has four splayed aromatic rings coming off of the cyclobutane ring. As expected, the *rctt*-stereochemistry in *hh*-PP results in two acute (76.5 and 81.5°) and two obtuse (96.7 and 117.1°) bond angles between the nearest neighboring aromatic rings and the central cyclobutane rings (Figure S1b). Importantly, the bond angle between the two 4-pyridyl groups within *hh*-PP was determined to be acute with a value of 76.5° (Figure 3). Each of the two ordered C_6_I_2_Cl_4_ molecules are found to engage in I⋯N halogen bonds (I⋯N·2.904(7) and 2.963(8) Å C-I⋯N 166.7(3) and 174.4(3)°) with 4-pyridyl rings on *hh*-PP. As a result of the 1,2-position for the halogen-bond acceptors on the cyclobutane, namely the 4-pyridyl group, the subsequent solid has a zigzag topology, forming a one-dimensional chain (Figure 3). The ability to control the regiochemistry (i.e., 1,2- or 1,3-position) of the halogen-bond acceptor groups on these various cyclobutane-based photoproducts ultimately controlled the topology of each solid, since the halogen-bond donor C_6_I_2_Cl_4_ acts as a linear spacer in each example.

**Figure 4:**
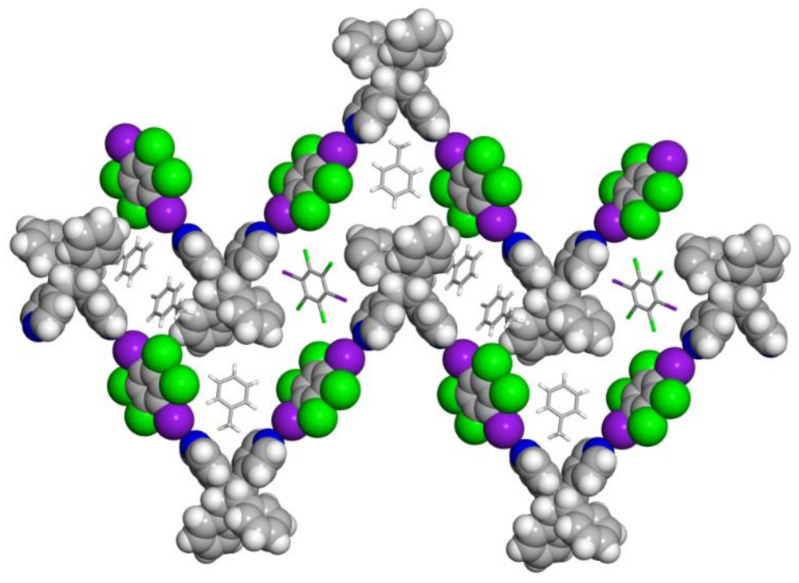
X-ray structure of 3(C_6_I_2_Cl_4_)·2(*hh*-PP)·4(toluene) illustrating the tongue and groove pattern between neighboring zigzag chains. The halogen-bonded zigzag chains are shown in a space-filled style. The observed disorder in C_6_I_2_Cl_4_ and toluene were removed for clarity. Grey ball-C atom, white ball-H atom; green ball-Cl atom; purple ball-I atom; blue ball-N atom.

Neighboring zigzag chains pack in a tongue and groove pattern that generates various void spaces throughout the crystal, which is filled with both the disordered C_6_I_2_Cl_4_ and two crystallographically unique toluene molecules (Figure 4). In particular, the disordered toluene is found in an irregular quadrilateral cavity nearest to the bridging 4-pyridyl groups. This quadrilateral cavity contains four different edge lengths that vary between 10.36 and 12.00 Å. The disordered C_6_I_2_Cl_4_ is found within a parallelogram cavity with edge lengths of 11.62 and 12.00 Å along with bond angles of 82.70 and 97.30°. Lastly, the ordered toluene is also found within a parallelogram cavity with slightly different parameters, namely 10.36 and 11.37 Å for the edge lengths and 70.76 and 109.24° for the bond angles. All of these edge lengths and bond angles were calculated based upon the centroid of the cyclobutane ring and bridging C_6_I_2_Cl_4_.

**Figure 5:**
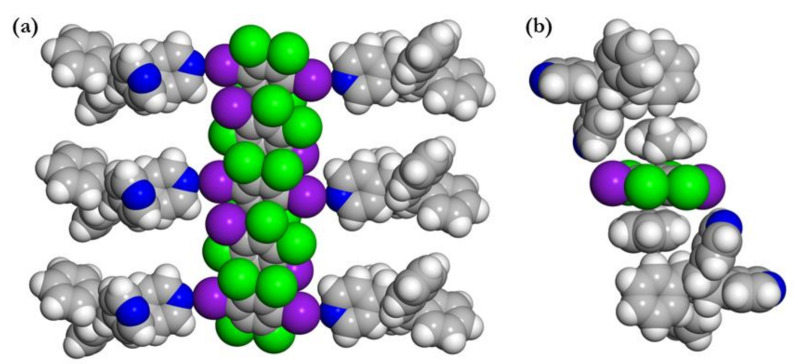
X-ray structure of 3(C_6_I_2_Cl_4_)·2(*hh*-PP)·4(toluene): (**a**) the infinite homogeneous stacking arrangements of C_6_I_2_Cl_4_ along the crystallographic *a*-axis and (**b**) the three-component heterogeneous stacking of C_6_I_2_Cl_4_ and equivalent toluene molecules. Grey ball-C atom, white ball-H atom; green ball-Cl atom; purple ball-I atom; blue ball-N atom.

The disordered C_6_I_2_Cl_4_ is found to stack in a homogeneous and offset face-to-face orientation with bridging C_6_I_2_Cl_4_. This π⋯π stacking arrangement generates an infinite column of C_6_I_2_Cl_4_ molecules that extends along the crystallographic *a*-axis (Figure 5a). The centroid-to-centroid distance between these C_6_I_2_Cl_4_ molecules was measured to be 4.72 Å. Lastly, the ordered toluene molecule is found to engage in heterogeneous and face-to-face π-π stacking with a bridging C_6_I_2_Cl_4_ (Figure 5b). Unlike before, this stacking arrangement forms a three-component stack where the C_6_I_2_Cl_4_ is found in the middle and surrounded by two equivalent toluene molecules with a centroid-to-centroid distance of 3.61 Å.

## 3. Materials and Methods

### 3.1. Materials

The reactant 4-stilbazole (4-SB) along with sodium hydroxide as well as the solvents toluene, ethanol, and chloroform were all purchased from Sigma-Aldrich Chemical (St. Louis, MO, USA) and used as received. The halogen-bond donor 1,4-diiodoperchlorobenzene (C_6_I_2_Cl_4_) was synthesized by a previously reported method [13]. The template 4,6-dibromoresorcinol (4,6-diBr res) was also prepared by a previously reported method [14]. All crystallization studies were performed in 20 mL scintillation vials.

### 3.2. General Methods

The [2 + 2] cycloaddition reactions were all conducted using UV-radiation from a 450 W medium-pressure mercury lamp in an ACE Glass photochemistry cabinet. Each solid was removed from its scintillation vials and placed between a pair of Pyrex glass plates for irradiation. The overall yield for the photoreaction was determined by using ^1^H NMR spectroscopy collected on a Bruker Avance 400 MHz spectrometer with DMSO-*d*_6_ as the solvent.

### 3.3. Formation of (C_6_I_2_Cl_4_)·(ht-PP)

The formation of the photoproduct *ht*-PP was achieved by a previously reported method [8]. In particular, co-crystals of (C_6_I_2_Cl_4_) 2(4-SB) were formed by dissolving 25.0 mg of C_6_I_2_Cl_4_ in 2.0 mL of toluene, which was then combined with a separate 2.0 mL toluene solution containing 19.4 mg of 4-SB (1:2 molar equivalent). After evaporation, the solid was removed from the vial and placed in the photoreactor for irradiation. After 30 h of UV exposure, a quantitative yield for the cycloaddition reaction to form *ht*-PP occurred as determined by ^1^H NMR [8]. The complete loss of the olefin peak on 4-SB at 7.57 ppm (Figure S2) along with the appearance and, in particular, the shape of the cyclobutane peak at 4.59 ppm (Figure S3) confirmed the quantitative photoreaction. The remaining solid was then dissolved in 3.0 mL of toluene and upon slow evaporation yielded single crystals suitable for X-ray diffraction within two days.

### 3.4. Formation of ·3(C_6_I_2_Cl_4_)·2(hh-PP)·4(toluene)

The formation of *hh*-PP was also achieved by a previously reported method [9]. Single crystals of the photoreactive solid (4,6-diBr res)·2(4-SB) were formed by dissolving 50.0 mg of 4-SB in 2.0 mL of ethanol, which was then combined with a separate 2.0 mL ethanol solution containing 24.7 mg of 4,6-diBr res (2:1 molar equivalent). After evaporation of most of the solvent, the resulting crystals were removed from the vial and then placed in the photoreactor for irradiation. After 20 h of UV exposure, a quantitative yield for the cycloaddition reaction was reached to form *hh*-PP as determined by ^1^H NMR. Again, the complete loss of the olefin peak on 4-SB at 7.57 ppm (Figure S2) along with the appearance of the cyclobutane peak at 4.59 ppm (Figure S4) confirmed the quantitative yield for this photoreaction. The difference in the shape of the cyclobutane peak confirmed the change in the regiochemistry to *hh*-PP [9]. An extraction using 5.0 mL of a 0.20 M sodium hydroxide solution was used to remove the 4,6-diBr res from *hh*-PP, which was separated by three aliquots of 10.0 mL of chloroform. The chloroform was removed under vacuum, which resulted in pure *hh*-PP. The co-crystal 3(C_6_I_2_Cl_4_)·2(*hh*-PP)·4(toluene) was achieved by combining 19.4 mg of *hh*-PP in 2.0 mL of toluene with a 2.0 mL toluene solution containing 25.0 mg of C_6_I_2_Cl_4_ (1:1 molar equivalent). The solution was allowed to slowly evaporate and after two days crystals suitable for X-ray diffraction were realized.

### 3.5. Single-Crystal X-ray Diffraction

X-ray data were collected on a Rigaku XtaLAB Synergy-*i* Kappa diffractometer equipped with a PhotonJet-*i* X-ray source to generate Cu Kα radiation. Crystals were transferred from the vial and placed on a glass slide in type NVH immersion oil by Cargille. Each crystal was mounted on a MiTeGen 50 μm MicroLoop and then transferred to the diffractometer where it was placed under a cold nitrogen stream at 100 K. Intensity data were corrected for Lorentz, polarization, and background effects using CrysAlisPro. A numerical absorption correction was applied based on a Gaussian integration over a multifaceted crystal and followed by a semi-empirical correction for adsorption. The program *SHELXT* [15] was used for the initial structure solution and *SHELXL* [16] was used for refinement of the structure. Both of these programs were utilized within the OLEX2 software [17]. Hydrogen atoms bound to carbons were geometrically constrained using the appropriate AFIX commands.

## 4. Conclusions

In this contribution, we report the ability to control the resulting topology of a pair of halogen-bonded extended networks based upon two regioisomers of a photoproduct formed in the organic solid state. This topological control was accomplished by varying the position of the halogen-bond accepting 4-pyridyl group around the cyclobutane ring. In particular, the *ht*-PP yielded a linear chain due to the divergent nature of the accepting groups, while the *hh*-PP resulted in a zigzag structure based upon its convergent nature. Currently, the incorporation of these photoproducts with other halogen- and hydrogen-bonding linkers is being investigated.

## Figures and Tables

**Table 1:** Crystallographic and refinement parameters for the extended networks (C_6_I_2_Cl_4_)·(*ht*-PP) and 3(C_6_I_2_Cl_4_)·2(*hh*-PP)·4(toluene).

Extended Network	(C_6_I_2_Cl_4_)·(*ht*-PP)	3(C_6_I_2_Cl_4_)·2(*hh*-PP)·4(toluene)
Formula	C_32_H_22_Cl_4_I_2_N_2_	C_49_H_38_Cl_6_I_3_N_2_
Formula Mass (g·mol^−1^)	830.11	1248.21
Crystal system	triclinic	triclinic
Space group	Pī	Pī
*a* (Å)	10.4767(2)	9.43028(16)
*b* (Å)	10.7864(2)	16.7708(2)
*c* (Å)	15.3827(2)	17.3759(3)
*α* (°)	106.830(1)	62.5359(16)
*β* (°)	93.005(1)	79.0112(16)
*γ* (°)	112.840(1)	74.7415(14)
*Z*	2	2
*V* (Å3)	1506.42(5)	2344.69(8)
*ρ*calcd (g·cm^−3^)	1.830	1.768
T (K)	100	100
μ (mm^−1^)	19.861	19.134
*F*(000)	804.0	1214.0
Radiation Source	Cu Kα	Cu Kα
Reflections collected	26,382	55,666
Independent reflections	5504	14,028
Data/restraints/parameters	5504/0/361	14,028/484/640
*R*_int_	0.0569	0.0422
*R*_1_ (*I* ≥ 2σ(*I*))	0.0325	0.0740
*wR* (*F*^2^) (I ≥ 2σ(I))	0.0852	0.2187
R^1^) (all data)	0.0342	0.0848
wR (F^2^) (all data)	0.0865	0.2377
Goodness-of-net on *F*^2^	1.036	1.029
CCDC deposition number	2078149	2078150

## Data Availability

The data presented in this study are available in the article and in the Supplementary Material. CIFs are openly available in www.ccdc.cam.ac.uk/data_request/cif.

## Supplementary Materials

The following are available online, Figure S1: Acute and obtuse angles within the photoproducts, Figure S2: ^1^H NMR of 4-SB, Figure S3: ^1^H NMR of (C_6_I_2_Cl_4_)·(*ht*-PP), Figure S4: ^1^H NMR of (*hh*-PP).

## Sample Availability

Samples of the compounds are not available from the authors.
