# Supplementary material for: Controlling Topology within Halogen-Bonded Networks by Varying the Regiochemistry of the Cyclobutane-Based Nodes
Source: Molecules. 2021 May 25;26(11):3152. doi: 10.3390/molecules26113152 (PMC8197507; doi:10.3390/molecules26113152)
Supplement: Supplementary file 1 [file molecules-26-03152-s001.zip › molecules-1209359-SI.pdf]

Supporting Information

for

Controlling Topology within Halogen-Bonded Networks by Varying the Regiochemistry of the Cyclobutane-based Nodes

Taylor J. Dunning, Daniel K. Unruh, Eric Bosch, and Ryan H. Groeneman

Department of Biological Sciences, Webster University, St. Louis, MO, 63119, USA

Department of Chemistry and Biochemistry, Texas Tech University, Lubbock, TX, 79409, USA

Department of Chemistry, Missouri State University, Springfield, MO, 65897, USA

Figure S1: Acute and obtuse angles within the photoproducts Page 2

Figure S2: ^{1}H NMR spectrum of **4-SB** Page 3

Figure S3: ^{1}H NMR spectrum of ($\mathbf{C_6I_2Cl_4}$)•(***ht*-PP**) Page 4

Figure S4: ^{1}H NMR spectrum of (***hh*-PP**) Page 5

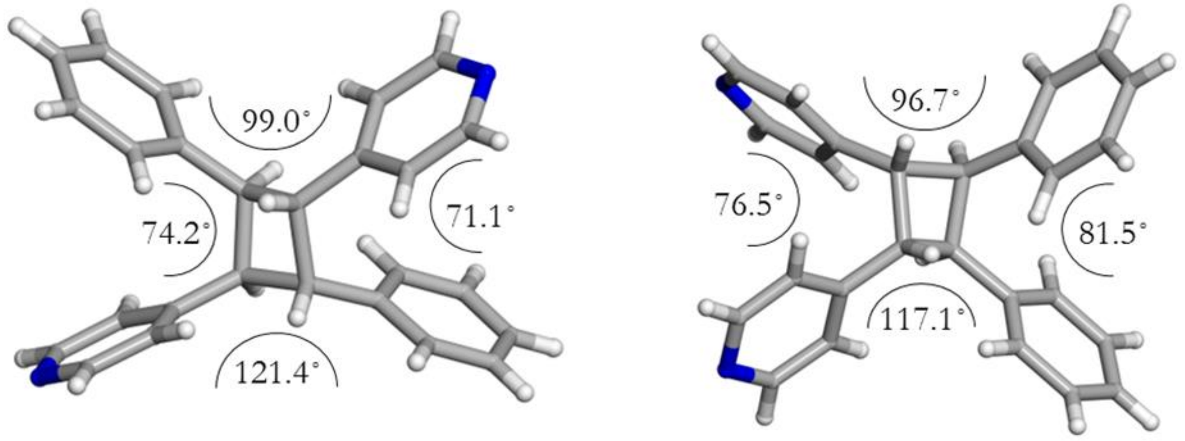

(a) **(b)**

Figure S1: X-ray structure illustrating the acute and obtuse angles observed within the different photoproducts (a) ***ht*-PP** and (b) ***hh*-PP**.

^{1}H NMR Spectroscopic Data

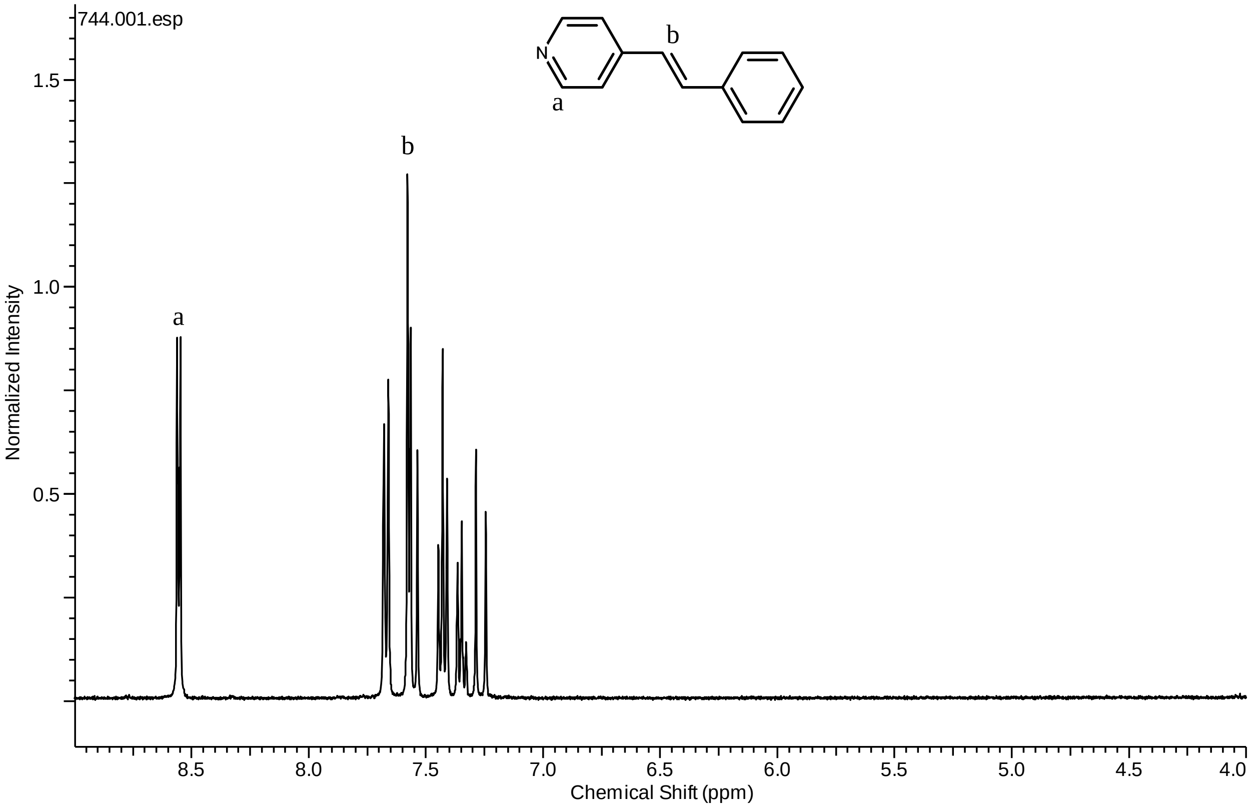

Figure S2: ^{1}H NMR spectrum of **4-SB** (400 MHz, DMSO-d_6).

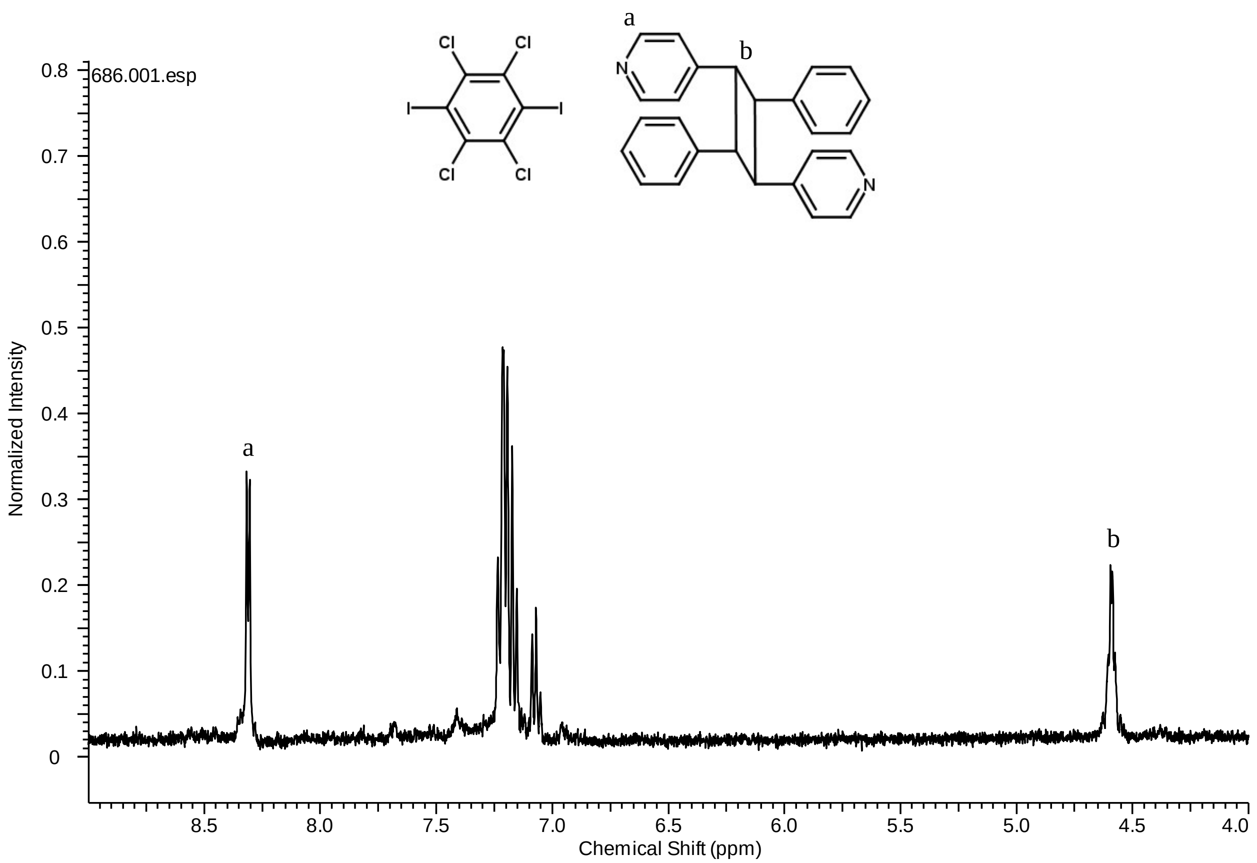

Figure S3: ^{1}H NMR spectrum of (**$C_6I_2Cl_4$**)•(***ht*-PP**) (400 MHz, DMSO-d_6).

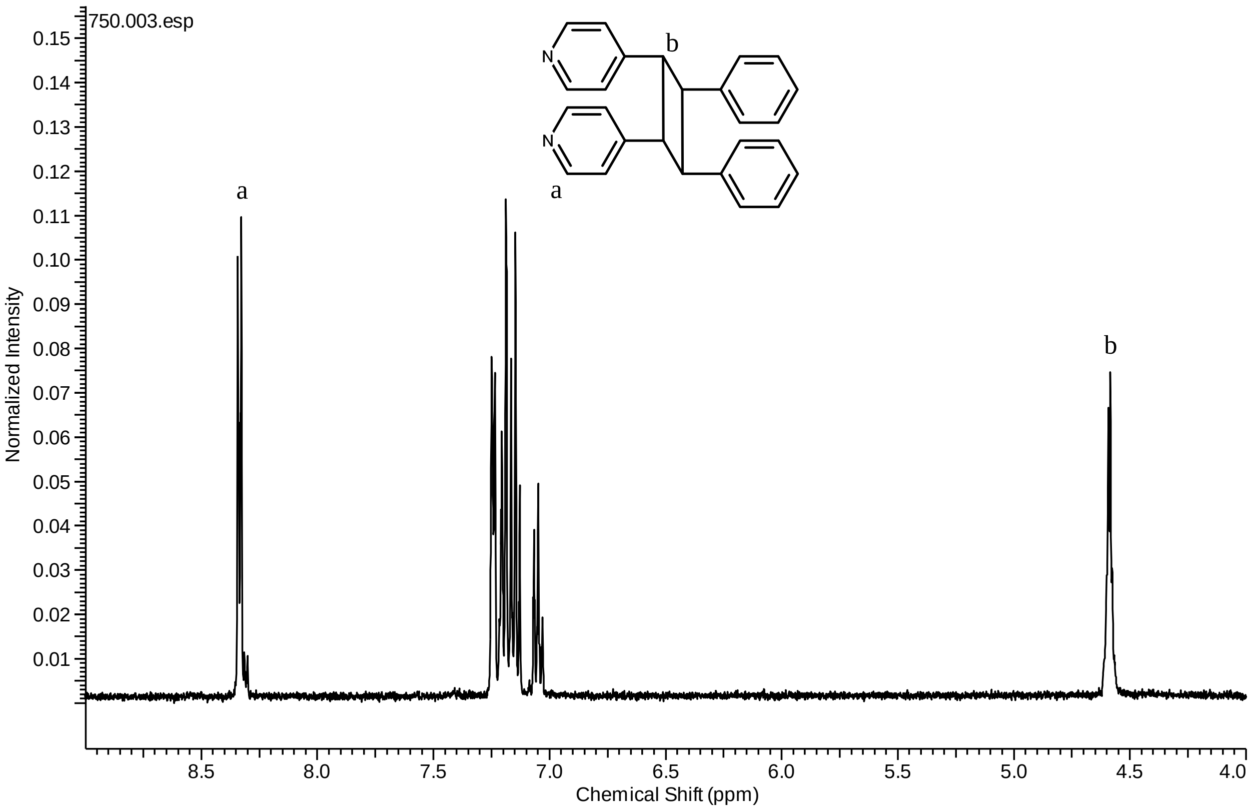

Figure S4: ^{1}H NMR spectrum of (***hh*-PP**) (400 MHz, DMSO-d_6).
